# Instant-SFH: Non-Iterative Sparse Fourier Holograms Using Perlin Noise

**DOI:** 10.3390/s24227358

**Published:** 2024-11-18

**Authors:** David Li, Susmija Jabbireddy, Yang Zhang, Christopher Metzler, Amitabh Varshney

**Affiliations:** 1 Department of Computer Science, University of Maryland, College Park, MD 20742, USA; jsreddy@umd.edu (S.J.); metzler@umd.edu (C.M.); varshney@umd.edu (A.V.); 2 Department of Electrical and Computer Engineering, University of Maryland, College Park, MD 20742, USA; yzhangdd@terpmail.umd.edu

**Keywords:** holography, Fourier holograms, Perlin noise

## Abstract

Holographic displays are an upcoming technology for AR and VR applications, with the ability to show 3D content with accurate depth cues, including accommodation and motion parallax. Recent research reveals that only a fraction of holographic pixels are needed to display images with high fidelity, improving energy efficiency in future holographic displays. However, the existing iterative method for computing sparse amplitude and phase layouts does not run in real time; instead, it takes hundreds of milliseconds to render an image into a sparse hologram. In this paper, we present a non-iterative amplitude and phase computation for sparse Fourier holograms that uses Perlin noise in the image–plane phase. We conduct simulated and optical experiments. Compared to the Gaussian-weighted Gerchberg–Saxton method, our method achieves a run time improvement of over 600 times while producing a nearly equal PSNR and SSIM quality. The real-time performance of our method enables the presentation of dynamic content crucial to AR and VR applications, such as video streaming and interactive visualization, on holographic displays.

## 1. Introduction

Holographic displays are a promising upcoming display technology with the ability to display 3D content by reconstructing the wavefield of a complete 3D scene. For AR and VR devices, holographic displays also have the potential to resolve the vergence–accommodation conflict, which leads to fatigue and virtual reality sickness [1,2,3]. Current holographic displays are prototyped using spatial light modulators (SLMs) based on micro-electro-mechanical systems (DMD or MEMS) or liquid crystal (LCD or LCoS) technology supporting phase-only holograms. Future holographic displays promise control of both amplitude and phase with dual liquid–crystal panels [4], metasurfaces [5,6,7,8], or nanophotonic phased arrays (NPAs) [9,10,11,12].

To address the high power consumption of large-scale NPA-based holographic displays, Jabbireddy et al. [13] propose using sparse holograms, complex holograms with zero amplitude across a large fraction of the hologram–plane pixels. Sparse holograms reduce power consumption by activating a sparse array of active antennas. The hologram layout, phase, and amplitude are computed using a Gaussian-weighted Gerchberg–Saxton (GGS) algorithm for a given target image. By adding a Gaussian weighted amplitude to the Gerchberg–Saxton algorithm, their method redistributes energy in the hologram plane, concentrating it in a few pixels and enabling high sparsity levels while maintaining the image quality of the complete non-sparse image. Due to the iterative nature of Gerchberg–Saxton, GGS takes over 2 s to generate the sparse hologram for a single channel on an RTX 2080 GPU. This limitation prevents sparse holograms from being used for interactive applications which encompass most AR and VR use cases such as data visualization [14], virtual collaboration [15], and live captioning [16].

Non-iterative methods in computer-generated holography, either conventional or learned ones, could help improve the speed of hologram generation. Traditionally, the Gerchberg–Saxton algorithm [17] is used for phase-retrieval applications, such as computing the hologram–plane phase for phase-only holograms. As an alternative to Gerchberg–Saxton, Pang et al. [18] propose a non-iterative method that combines a quadratic image–plane phase with an error diffusion technique. However, this method is unsuitable for generating sparse holograms, which concentrate the amplitude in a few hologram pixels rather than diffusing it throughout all the pixels.

To enable sparse holograms for dynamic and interactive applications, in this paper, we examine how the Gaussian-weighted Gerchberg–Saxton (GGS) algorithm generates sparse holograms. We observe that each iteration of the GGS algorithm smooths out the image–plane phase, converging to a primarily low-frequency phase. Based on this observation, we propose to replace the random phase initialization of GGS with a low-frequency initial phase based on Perlin noise [19,20]. We show that, by selecting a sufficiently low frequency, a Perlin-noise-based phase initialization directly results in a converged solution for GGS, allowing us to instantly generate a sparse hologram.

**Figure 1:**
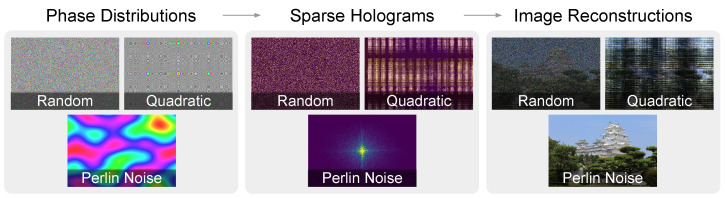
On the (**left**), we show a comparison between the random phase, the quadratic phase [18], and Perlin noise [19,20]. In the (**middle**), we show the amplitudes of 80% sparse holograms constructed using each of these image–plane phase distributions. On the (**right**), we show images reconstructed from the sparse holograms for each of these image–plane phase distributions.

Figure 1 shows a visual overview of our algorithm results with a random phase, a quadratic phase, and a Perlin-noise-based phase. We examine how the image–plane phase distribution affects the amplitude distribution of Fourier holograms. We discover that using a low-frequency Perlin noise allows us to generate sparse Fourier holograms (visualized at 80% sparsity) with an over 600× speedup over the current method.

In summary, the contributions of our paper are as follows:

- We examine how the Gaussian-weighted Gerchberg–Saxton algorithm optimizes sparse holograms and discover that the frequency of the image–plane phase decreases until convergence.
- We propose to use low-frequency Perlin noise as the image–plane phase, allowing us to create sparse holograms in about one millisecond.
- We explore the relationship between the frequency of the image–plane phase and the reconstruction quality of sparse holograms.

## 2. Background and Related Work

Our work builds upon the existing research in computer-generated holography. First, we present some background on the sparse holograms. Then, we provide a brief overview of recent work in holographic displays. Finally, we discuss related work in Gerchberg–Saxton phase retrieval.

### 2.1. Sparse Holograms

Computer-generated holograms (CGHs) can be classified into three types: amplitude-only holograms, phase-only holograms, and complex holograms. Most existing work in CGHs focuses on generating phase-only holograms to accommodate the commercially available phase-only spatial light modulators found in existing holographic display setups. Our work focuses on sparse complex holograms designed for future holographic display setups with independent control of amplitude and phase for each hologram pixel.

**Figure 2:**
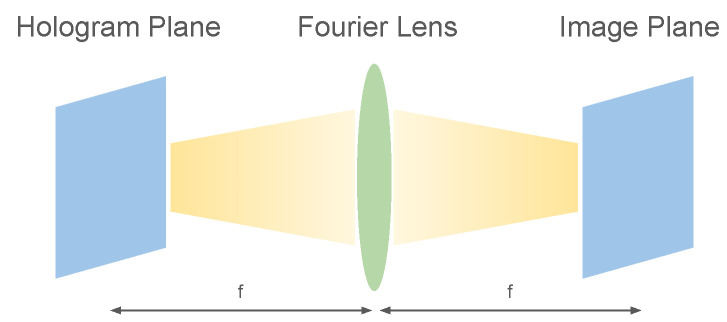
Visual overview of a Fourier hologram setup. On the hologram plane (**left**), a complex hologram with amplitude and phase is emitted from the hologram plane. The hologram passes through a Fourier lens (**middle**). On the image plane (**right**), a complete image is emitted.

**Figure 3:**
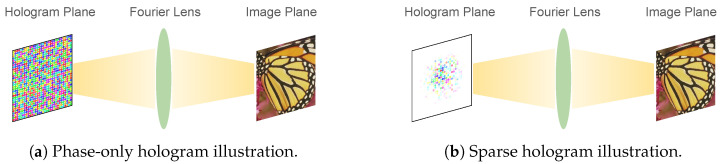
Illustrative comparison between a (**a**) phase-only hologram and a (**b**) sparse hologram. In (**a**), pixels on the hologram plane have equal amplitude (visualized as transparency) and differing phases (visualized as hue). In (**b**), only a subset of pixels are active with non-zero amplitude and differing phases. In both (**a**) and (**b**), a complete image can be produced on the image plane.

In a far-field Fourier hologram setup, the hologram plane and the image plane are related by a Fourier transform, as shown in Figure 2. Since a far-field hologram undergoes a Fourier transform, we can use a hologram with sparsely activated pixel to display a complete image. As illustrated in Figure 3b, a sparse hologram is a hologram where only a subset of the hologram–plane pixels are activated with a non-zero amplitude.

Whereas phase-only holograms, illustrated in Figure 3a, are directly applicable to the current generation of phase-only spatial light modulators (SLMs), sparse holograms are intended to address upcoming SLMs that promise control of both amplitude and phase. Jabbireddy et al. [13] explore sparse holograms targeting nanophotonic phased arrays (NPAs) [9,10,11,12], where each antenna, or ‘pixel’, emits amplitude and phase independently. Under such a setup, the usage of each hologram pixel directly contributes to the power consumption and heat generation of the holographic display.

We note that this use of sparsity differs from Shi et al. [21], which uses the sparsity of light fields in the angular domain for light field reconstruction. Most existing methods such as Maimone et al. [22] and Peng et al. [23] are targeted towards generating real-time phase-only CGH rather than sparse CGH.

### 2.2. Holographic Displays and Algorithms

Holographic displays generate images through the controlled diffraction and interference of coherent light. Unlike traditional displays, holographic displays reconstruct a wavefront of the scene by controlling the phase of the emitted light. Holographic displays are built using spatial light modulators (SLMs). Current SLMs are built upon liquid crystal-based (LCD) or micro-electro-mechanical system (MEMS) technology. Existing SLMs typically only offer phase-only modulation and can also be referred to as phase spatial light modulators (PLMs). For complex holograms which have both amplitude and phase, researchers are in the process of developing two-phase holograms [24], metasurfaces [6,7], and nanophotonic phased arrays [9,10].

Digital holography falls into a class of problems known as inverse problems [25,26,27], since they aim to recover the hologram amplitude and phase which generates the observed target image. Two common types of holograms are Fresnel and Fourier holograms. Fresnel holograms are used in near-field holographic displays. Our paper focuses on Fourier holograms, which are used in far-field holographic displays.

### 2.3. Gerchberg–Saxton Phase Retrieval

Conventional spatial light modulators (SLMs) can only control either the amplitude or phase of the coherent light. In the case of phase-only SLMs, the Gerchberg–Saxton (GS) algorithm [17] is often used to compute the phase in the hologram plane with strict amplitude constraints in the hologram and image planes. Existing research has focused on improving the convergence performance and robustness of the GS algorithm for generating phase-only holograms.

For phase-only Fourier holograms, Chen et al. [28,29] develop a weighted constraint iterative algorithm (WCIA). Given target amplitude $A_{t}$, reconstructed amplitude $A_{r}$, and input amplitude $A_{k}$, WCIA uses an adaptive over-compensation in their amplitude constraint, $|A_{con}\left|=\right|A_{k}\left|(\right|A_{t}\left|/\right|A_{r}{\left|\right)}^{\beta_{k}}$, in the signal region. Reducing the difference between the reconstructed amplitude and the new amplitude in the image plane allows for better amplitude uniformity in the hologram which enhances the convergence of Gerchberg–Saxton. Pang et al. [18] propose a non-iterative method to generate phase-only Fourier holograms using a quadratic phase ($\varphi =am^{2}+bn^{2}$), which represents illumination with a point light source. Building upon WCIA, Wu et al. [30] develop an adaptive weighted Gerchberg–Saxton (AWGS). AWGS replaces the weighted ratio in the amplitude constraint with $e^{A_{t}-A_{r}}$ to achieve more stable convergence. Furthermore, they propose initializing the phase in the target domain with an approximation to a quadratic phase. With both of these techniques, AWGS can generate phase-only Fourier holograms with stable convergence, fewer artifacts, and a PSNR that is 4.8 dB higher.

Thimons and Wittle [31] investigate the Gerchberg–Saxton algorithm under different conditions, including applying a low-frequency filter to the input magnitude, multiplying the input magnitude with Gaussian, and implementing a constant initial phase. In their experiments, they found that, when one set of magnitudes is non-centrosymmetric, using constant phase initialization leads to more stable and accurate convergence in far fewer iterations. Cruz-López and González-Velázquez [32] analyze the effects of several random phase distributions on Fourier and Fresnel hologram reconstruction. While we also examine the phase distribution, our method uses Perlin noise to generate sparse complex holograms rather than amplitude-only or phase-only holograms.

In contrast to these existing works, which develop algorithms for phase-only holograms and existing phase-only SLM hardware, our work is focused on the constraint of sparsity for future complex SLM hardware. To the best of our knowledge, we believe Instant-SFH is the first to use Perlin noise for constructing sparse holograms and the first to generate sparse holograms in real time with equivalent quality to those generated by GGS.

## 3. Method

### 3.1. Gaussian-Weighted Gerchberg–Saxton

In a Fourier hologram setup, an SLM produces a complex-valued source hologram $E_{s}=Se^{i\phi_{S}}$ with amplitude *S* and phase $\phi_{S}$. The source hologram propagates through free space or a Fourier lens to form a target far-field image $E_{t}=Te^{i\phi_{T}}$. At the far-field image plane, only the intensity $I\propto T^{2}$ is perceptible to the viewer. Jabbireddy et al. [13] propose the Gaussian-weighted Gerchberg–Saxton (GGS) algorithm to generate sparse amplitude and phase layouts for Fourier holograms. GGS leverages the Gerchberg–Saxton algorithm while modulating the amplitude in the hologram and image planes. However, unlike in traditional phase-retrieval applications, GGS does not apply a strict amplitude constraint in the hologram plane. Instead, at each iteration *i*, the amplitude constraint $A_{con}$ used is a element-wise product of a Gaussian weight map W and the current amplitude $|A_{i}|$ in the hologram plane:

(1) $$A_{con}=W⊙\left|A_{i}\right|$$

Upon convergence, or after 1000 iterations, the amplitudes in the hologram plane are truncated to the desired sparsity level, resulting in a sparse layout for a Fourier hologram with amplitude and phase.

In their experiments, Jabbireddy et al. [13] observe that each iteration of GGS takes about 3.5 ms for a single channel. In total, their end-to-end pipeline takes 2.26 s per image channel on average. Without the ability to run in real time, GGS is limited to generating holograms of things that can be pre-computed offline, such as static images and text. This limitation prohibits using sparse holograms in interactive applications and makes it difficult to view even videos, which would require pre-computing and storing a sequence of sparse holograms.

**Figure 4:**
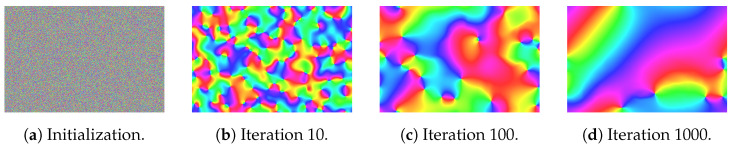
Image plane phase of Gaussian-weighted Gerchberg–Saxton (GGS) visualized with angles directly mapped to color hues visualized at initialization (**a**), after 10 GGS iterations (**b**), after 100 GGS iterations (**c**), and after 1000 GGS iterations (**d**).

**Figure 5:**
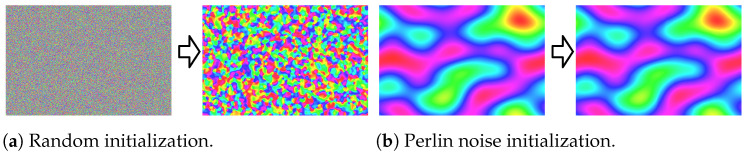
Image–plane phase visualizations at initialization and after a single GGS iteration. In (**a**), we show a random initial phase and the phase after a single iteration. In (**b**), we show a Perlin-noise-based initial phase the phase after a single iteration.

A real-time method would enable sparse holograms to be used in far more scenarios such as video streaming, data visualization, and other interactive applications. With the goal of real-time sparse holograms in mind, we first examine how GGS generates sparse holograms. The standard Gerchberg–Saxton algorithm enforces a strict amplitude constraint in both the image plane and hologram plane. GGS uses a more relaxed constraint by modulating the amplitude in the hologram plane instead of replacing it in each iteration. However, the amplitude is still strictly enforced in the image plane. Since the Fourier transform is an invertible transformation between holograms and images, the amplitude constraint in the hologram plane must be reflected in the image–plane phase. We visualize how the image–plane phase changes in Figure 4 and Figure 5a. In Figure 5a, we see that the phase changes significantly at the first iteration. This is expected of a non-converged phase. As GGS progresses through 1000 iterations in Figure 4, the image–plane phase quickly becomes smoother, consisting primarily of low-frequency components.

### 3.2. Perlin Noise

Since GGS converges to a low-frequency phase distribution, we hypothesize that initializing with a low-frequency phase will result in faster convergence. To initialize the image–plane phase with a low-frequency distribution, we propose the use of Perlin noise [19,20]. Perlin noise is a band-limited noise function that allows direct control over the frequency content and can be made periodic with a proper selection of gradient vectors. This property is not available in other types of noise such as Gaussian noise. In computer graphics, Perlin noise is commonly used to generate procedural textures and terrain [19,33,34].

Perlin noise [19,20] is generated by enumerating a low-resolution 2D grid with unit-length 2D vectors, known as gradient vectors. Next, we enumerate a high-resolution 2D image grid mapping to the low-resolution grid at a particular frequency. For each image pixel, offset vectors to the four nearest low-resolution grid points are computed. The dot products between gradient and offset vectors are computed at each grid point. Finally, a smooth interpolation is applied across the four dot product values to produce a smooth noise image. We scale the resulting Perlin noise output to an angle between 0 to 2π. Implementations of Perlin noise are available online (Perlin noise: https://github.com/pvigier/perlin-numpy, accessed on 21 April 2023).

**Figure 6:**
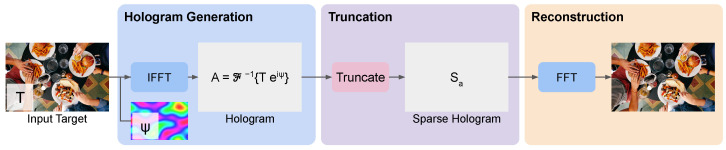
Overview of our instant sparse Fourier hologram (Instant-SFH) evaluation pipeline with three components: hologram generation, truncation, and reconstruction. In hologram generation, the target amplitude is augmented with a Perlin noise phase and converted to a complex hologram using inverse FFT. In truncation, the complex hologram is converted to a sparse hologram by zeroing out pixels at the lowest amplitude values. In reconstruction, we apply far-field propagation by taking an FFT to reconstruct the image.

**Figure 7:**
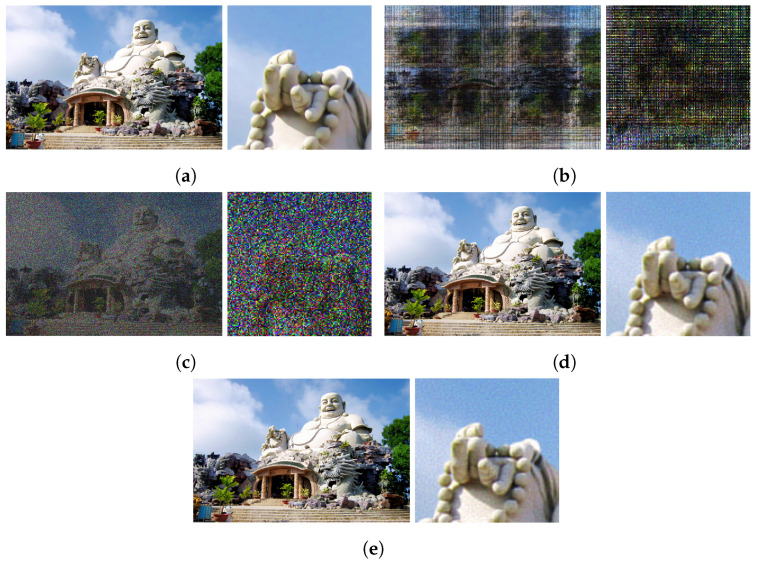
Comparison of 80% sparse hologram reconstructions using several methods. The original image is first shown in (**a**) as a reference. Then for each method, we show the full image and a zoomed-in crop. (**a**) Original image; (**b**) sparse hologram reconstruction with quadratic phase, (≈1 ms); (**c**) sparse hologram reconstruction with random phase, (≈1 ms); (**d**) sparse hologram reconstruction with random phase and 1000 GGS iterations (≈600 ms); (**e)** sparse hologram reconstruction with Perlin-noise-based phase (≈1 ms).

When replacing the random initial phase with a low-frequency Perlin-noise-based phase, we observe that successive GGS iterations do not significantly change the image–plane phase, as shown in Figure 5b, suggesting a converged solution. Hence, with Perlin noise, a single inverse FFT can be used to generate a sparse hologram without the need for GGS, as shown in Figure 6. Directly generating a sparse hologram with a high-frequency random phase [17] or quadratic phase [18] does not result in an acceptable reconstruction, as shown in Figure 7.

## 4. Experiments

We first examine the relationship between the frequency of the image–plane phase and the sparse Fourier hologram reconstruction quality. Next, we explore whether there is a relationship between the frequency of the image amplitude and the frequency of the image phase when generating sparse holograms. Finally, we evaluate the quality and performance of our instant sparse Fourier hologram method by comparing it to the Gaussian-weighted Gerchberg–Saxton method.

### 4.1. Implementation and Setup

Our experiments are conducted using an existing image dataset. Following Jabbireddy et al. [13], we use the X2 bicubic split of the DIV2K dataset (Data available at https://data.vision.ee.ethz.ch/cvl/DIV2K/, accessed on 21 April 2023) [35]. This dataset contains a total of 900 images, most of which have a landscape orientation with 1020×678 resolution. To ensure consistent quality and performance evaluation results, we rotate portrait images to landscape and resize and center crop all images to 1020×678. We evaluate our method using all 900 images. We implement our algorithm and baseline methods using PyTorch 2.0.1, which enables GPU-accelerated computations. Our experiments are run on a workstation with an Intel Core i7-12700K and an NVIDIA RTX 4090.

We first evaluate the performance and quality of our instant sparse Fourier hologram method (Instant-SFH). We compare our method to the existing offline GGS method [13], which uses a random initial phase and up to 1000 iterations for convergence. Note that the offline GGS method is not suitable for dynamic content such as streaming video or interactive 3D visualization. To quantitatively evaluate the reconstruction quality of our methods, we use two standard reference-based image quality metrics: the peak signal-to-noise ratio (PSNR) and the structural similarity metric (SSIM).

### 4.2. Frequency Ablation

Perlin noise [19,20] is generated by enumerating a grid with unit-length vectors and interpolating between grid values using dot products and linear interpolation. The sampling rate, or the ratio between image pixels and the underlying grid, represents the frequency of the noise generated. To determine the ideal frequency of Perlin noise to use for the image–plane phase, we evaluate how different frequencies affect the reconstruction quality. Specifically, we conduct experiments across 28 frequencies: 0,1,...,9,10,20,...,90,100,200,...,900. This value is the resolution of the underlying grid being sampled when generating Perlin noise. For frequency 0, we use a constant zero phase across the image.

**Figure 8:**
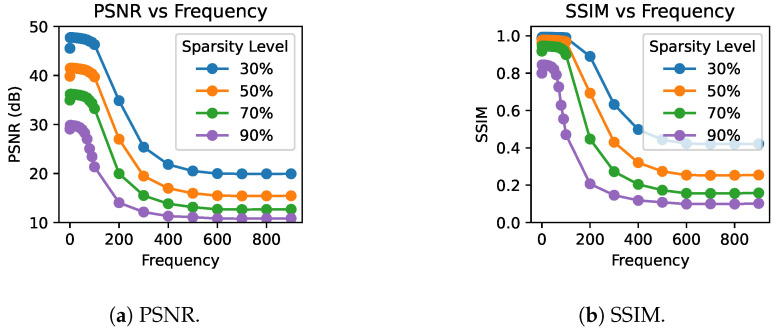
Quantitative evaluation of the sparse hologram reconstruction quality across Perlin noise frequency levels from 0 to 100 and sparsity levels from 30% to 90%.

**Figure 9:**
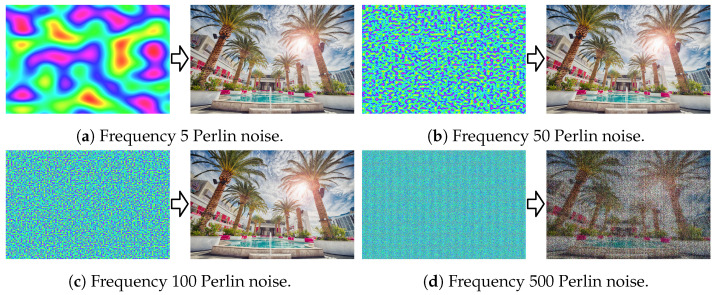
Qualitative ablation results showing the generated Perlin noise (left) and resulting 80% sparse hologram reconstruction (right) at Perlin noise frequency levels (**a**) 5, (**b**) 50, (**c**) 100, and (**d**) 500. Please zoom in to see the differences in details.

**Figure 10:**
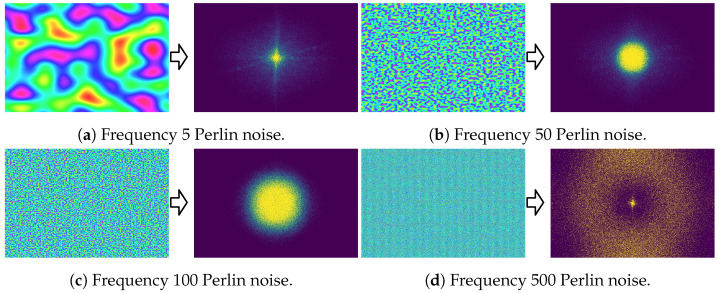
Qualitative ablation results showing the generated Perlin noise (left) and resulting 80% sparse hologram amplitudes (right) at Perlin noise frequency levels (**a**) 5, (**b**) 50, (**c**) 100, and (**d**) 500. The amplitude corresponds to the red channel of the image in Figure 9, where yellow pixels indicate amplitudes at or above 0.001.

Quantitative results averaged over all 900 images (28×900=25.2 K reconstructions) are shown in Figure 8. Qualitative results at a few frequency levels are shown in Figure 9 with corresponding hologram amplitudes visualized in Figure 10. Across all sparsity levels, we observe that the PSNR and SSIM quality decreases as the frequency of the Perlin noise increases. The PSNR and SSIM values are maximized at the lowest non-zero frequencies, specifically frequencies 1 to 10. Additionally, we observe that the higher the sparsity level, the more sensitive the reconstruction is to the phase frequency. For the majority of our evaluation, namely Section 4.4 and Section 4.5, we use a frequency of 3 for our Instant-SFH method.

### 4.3. Amplitude vs. Phase Frequency

Next, we examine whether there is a relationship between the frequency of the amplitude and the frequency of the phase in the image plane when generating sparse holograms. To control the frequency of the amplitude, we apply a Gaussian blur to the target image and conduct evaluations using different band-limited targets.

**Figure 11:**
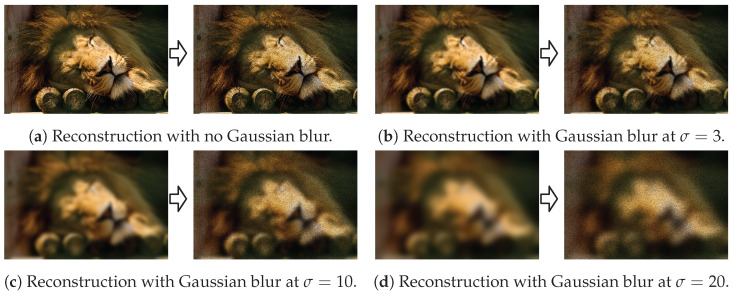
Qualitative results showing band-limited target images and resulting 80% sparse hologram reconstruction quality at frequency 200 Perlin noise.

**Figure 12:**
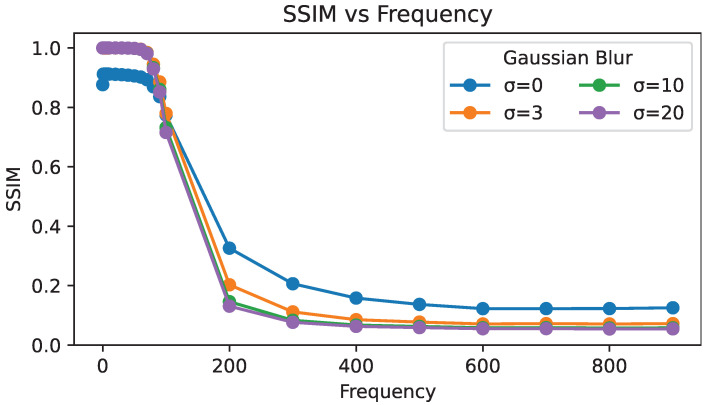
Quantitative evaluation with Gaussian blurred target images at 80% sparsity.

Qualitative results on a single image are shown in Figure 11, and quantitative results averaged over all images are shown in Figure 12. Gaussian-blurred images can be easily reconstructed with low-frequency phases, achieving a higher SSIM than normal images at the same sparsity level due to the lack of high-frequency details. With a high-frequency phase, speckling artifacts emerge in sparse hologram reconstructions. These artifacts are much more noticeable in low-frequency (e.g., blurred) regions of the target image that otherwise lack high-frequency details in the reconstruction. Hence, the SSIM of Gaussian-blurred image reconstructions falls below that of standard images.

### 4.4. Performance

First, we evaluate the performance of our method to determine whether interactive performance can be achieved by removing GGS. We present cumulative performance results over all three color channels in Table 1. Note that the results shown are rounded so run times for all stages may not add up to the total run time. On our workstation, we observe that offline GGS takes 664.4 ms, making it unsuitable for interactive scenarios. Using GGS in an interactive scenario would result in less than 2 FPS. Meanwhile, our instant sparse Fourier hologram method runs in 1.0 ms on average, a performance improvement of over 600×. Next, we benchmark our Instant-SFH method at higher resolutions to determine whether it is suitable for large-field-of-view applications. At 720p HD and 1080p full HD resolutions, our method runs in 1.2 ms and 4.0 ms, respectively, as shown in Table 2. The high performance of our method allows sparse holograms to take advantage of the high refresh rate available in holographic displays and enables high-refresh-rate and high-resolution content to be displayed.

### 4.5. Quality

**Figure 13:**
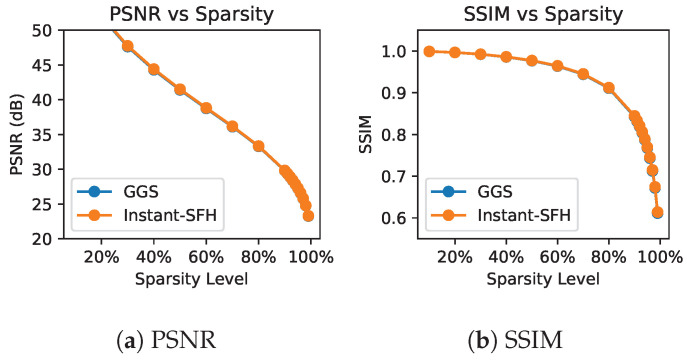
Quantitative comparison between GGS [13] and our Instant-SFH method. Note that GGS and Instant-SFH have overlapping results. Results are averaged over 900 images.

Finally, we compare the image reconstruction quality of GGS and Instant-SFH. Quantitative results for GGS and Instant-SFH are shown in Table 3. In the offline setup, where up to 1000 iterations of GGS can be computed, initializing with a random phase achieves very high PSNR and SSIM results even at 70% or 80% sparsity. With Perlin noise, our Instant-SFH method matches the PSNR and SSIM of GGS without requiring any iterations of GGS. Random phase [17] and quadratic phase [18] generate a sparse hologram with PSNR values around 11 dB, which is not acceptable. A constant zero phase yields a sparse hologram but with ≈1 dB worse PSNR results than low-frequency Perlin noise. Finally, optimized random phase (ORAP) [36], which involves transferring a phase distribution optimized for another image, achieves PSNR and SSIM scores slightly below that of GGS and Perlin noise. In Figure 13, our Instant-SFH method achieves PSNR and SSIM results consistent with GGS across all sparsity levels.

### 4.6. Optical Verification

**Figure 14:**
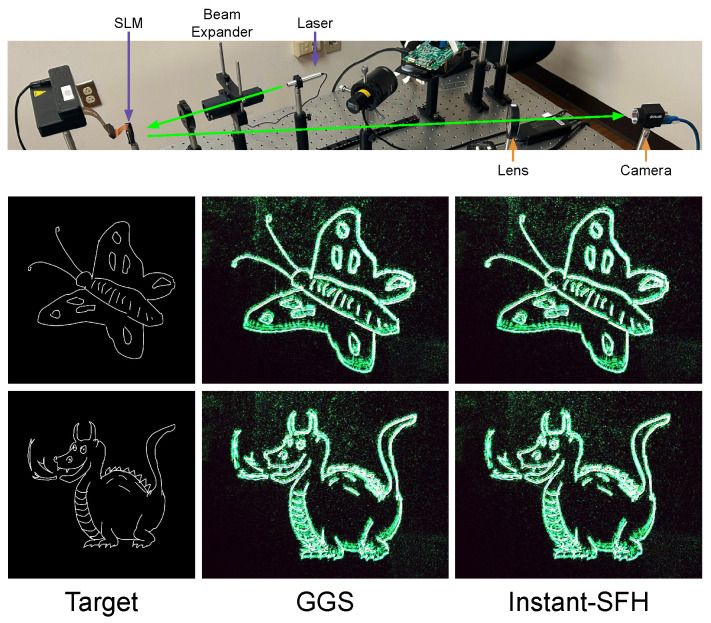
Optical verification setup and results. Sparse holograms (80% sparsity) from GGS and Instant-SFH are converted to phase-only holograms using double phase-amplitude coding and displayed on a phase-only SLM.The left column shows the target images. The center column shows images generated from GGS. The right column shows images generated from Instant-SFH.

In the absence of commercially available amplitude-phase SLMs, we use a phase-only SLM to optically verify that our Instant-SFH method achieves similar results to GGS. Specifically, we generate 80% sparse complex holograms using GGS and Instant-SFH. Both sets of sparse holograms are then converted to phase-only holograms using the double-phase amplitude coding (DPAC) [22,23,37] method and displayed on a phase-only SLM. While our method is broadly applicable to natural images, phase-only holograms produced by DPAC cannot encode high amplitude information at low frequencies [38]. Consequently, we opt to display sketches from the human sketch image dataset published by Eitz et al. [39] (Sketch Dataset: https://cybertron.cg.tu-berlin.de/eitz/projects/classifysketch/, accessed on 21 April 2023). Our setup consists of a Holoeye Leto SLM with 1080p resolution and 6.4 µm pixel pitch. Coherent light emitted by a 532 nm green laser is passed through a beam expander and is incident on the SLM. The modulated light forms a far-field image, which is captured by a 1024×1280 camera through a 200 mm lens. Our optical verification setup and results are shown in Figure 14. From our experiments, we see that Instant-SFH generates similar results to GGS despite only taking a fraction of the time. In this situation, we replace reconstruction with DPAC, which only takes 0.1 ms. We attribute the speckling artifacts to the DPAC conversion and environmental variables since they exist in both the results of GGS and Instant-SFH. As our objective is to show the quality similarity between our real-time method and offline GGS, we have not tuned the Perlin noise to improve the quality for this scenario.

## 5. Discussion

This paper focuses on Fourier holograms that display flat 2D images, similar to other recent holography papers [13,40]. To achieve the full benefits of holographic displays including accommodation and motion parallax, 3D content can be displayed using near-field holograms with multiple focal planes [41,42]. Since Instant-SFH runs at KHz rates, our method can be extended to display 3D by generating multiple 2D depth slices and time-multiplexing near-field 2D holograms generated with a Fresnel transform. Alternatively, the 3D method by Chakravarthula et al. [43] also applies whereby an eye-tracker is used to determine the depth of the foveal region based on the scene and a 2D Fresnel hologram is shown at the detected depth.

**Figure 15:**
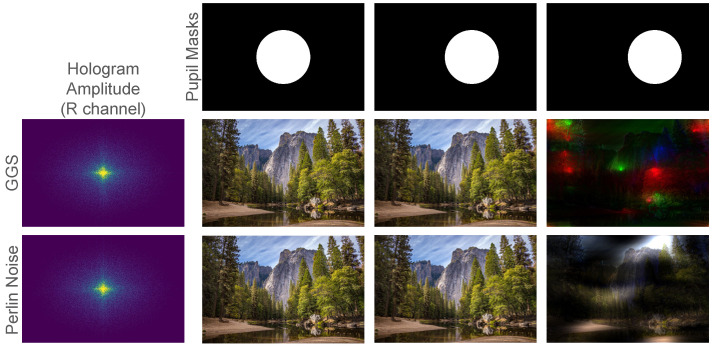
Eyebox comparison between GGS and Instant-SFH with Perlin-noise-based phase at 80% sparsity. The pupil mask (top row) shows the pupil moving progressively to the right. The center row shows images reconstructed from GGS. The bottom row shows images reconstructed from Instant-SFH using Perlin noise.

As observed by Chakravarthula et al. [40], a constant phase can lead to a smaller effective eye box as the light energy is concentrated in the small, low-frequency region. In Figure 15, we observe that Instant-SFH and GGS have a similar eyebox as both use smooth phases, prioritizing hologram quality over eyebox size. While outside the scope of this paper, our sub-ms run-times also open up the possibility of adaptively compensating for a moving eyebox. For example, in a pupil-tracked environment [44], one could pair Perlin noise with a few iterations of GGS and a pupil-weighted threshold [40] when determining the amplitudes at the SLM. Such compensation is only viable for algorithms, like ours, that can run at KHz rates.

Time-division multiplexing for multiple focal planes and dynamic eyebox adjustment require fast algorithms to achieve interactive frame rates. The millisecond run times of Instant-SFH open up the possibility of pairing such techniques with sparse holograms. Our evaluation performs FFT using a desktop NVIDIA RTX 4090 GPU. To deploy our algorithm on more portable form factors, we believe a digital signal processor with the ability to perform FFT in real time would be necessary to accommodate the requirements of Instant-SFH.

## 6. Conclusions

In this paper, we examine how sparse Fourier holograms can be adapted to view dynamic content such as videos and interactive applications. First, we examine how the Gaussian-weighted Gerchberg–Saxton (GGS) algorithm alters the image–plane phase. Next, we discover that using Perlin noise allows for the instantaneous generation of sparse Fourier holograms, eliminating the need for any iterations of GGS. Finally, we quantitatively evaluate how the frequency of the Perlin noise affects the hologram reconstruction quality across an existing dataset of 900 images and on band-limited images. Our Instant-SFH method achieves a performance improvement of over 600 times, opening up the possibility of using sparse holograms for dynamic content. We believe our method will broaden the use of upcoming holographic displays for dynamic applications.

## Figures and Tables

**Table 1:** Run time (ms) evaluation comparing running GGS with 1000 iterations and our Instant-SFH method at 1020×678 resolution. The results are the total run time for all three channels of the RGB images. Numbers in parentheses indicate standard deviation.

Method	Generate	Truncate	Reconstruct	Total
GGS	595.6	0.4	0.2	596.2
(std)	(1.0)	(0.1)	(0.0)	(1.0)
Instant-SFH	0.4	0.4	0.2	1.0
(std)	(0.1)	(0.1)	(0.0)	(0.1)

**Table 2:** Run time (ms) evaluation for our Instant-SFH method at 720p (1280×720) and 1080p (1920×1080) resolution. Numbers in parentheses indicate standard deviation.

Resolution	Generate	Truncate	Reconstruct	Total
1020×678	0.4	0.4	0.2	1.0
(std)	(0.1)	(0.1)	(0.0)	(0.1)
720p	0.4	0.5	0.3	1.1
(std)	(0.0)	(0.0)	(0.1)	(0.1)
1080p	1.8	0.9	1.2	3.8
(std)	(0.0)	(0.1)	(0.1)	(0.2)

**Table 3:** Quantitative comparison of GGS with 1000 iterations and Instant SFH with different image phases. Results are computed at 70% sparsity and averaged over 900 images. Numbers in parentheses indicate standard deviation. Bold numbers indicate the best results.

Method	Initial Phase	PSNR (dB)	SSIM
GGS	Random	36.12 (4.16)	0.944 (0.034)
Instant-SFH	Random	10.88 (2.29)	0.118 (0.064)
Instant-SFH	Quadratic	10.90 (2.18)	0.139 (0.070)
Instant-SFH	Zeros	34.98 (4.24)	0.918 (0.050)
Instant-SFH	ORAP	36.02 (4.15)	0.943 (0.035)
Instant-SFH	Perlin	**36.19** (4.21)	**0.945** (0.034)

## Data Availability

Our simulated experiments are conducted on the X2 bicubic split of the DIV2K dataset [35] available at https://data.vision.ee.ethz.ch/cvl/DIV2K/ (accessed on 21 April 2023). Our optical verification results are conducted on the sketch dataset by Eitz et al. [39] available at https://cybertron.cg.tu-berlin.de/eitz/projects/classifysketch/ (accessed on 21 April 2023).
